# Peptide-Folding Triggered Phase Separation and Lipid Membrane Destabilization in Cholesterol-Rich Lipid Vesicles

**DOI:** 10.1021/acs.bioconjchem.2c00115

**Published:** 2022-04-01

**Authors:** Johanna Utterström, Hanna M. G. Barriga, Margaret N. Holme, Robert Selegård, Molly M. Stevens, Daniel Aili

**Affiliations:** † Laboratory of Molecular Materials, Division of Biophysics and Bioengineering, Department of Physics, Chemistry and Biology, SE-581 83 Linköping, Sweden; ‡ Department of Medical Biochemistry and Biophysics, Karolinska Institutet, SE-171 77 Stockholm, Sweden; § Department of Materials, Department of Bioengineering and Institute of Biomedical Engineering, Imperial College London, London SW7 2AZ, U.K.

## Abstract

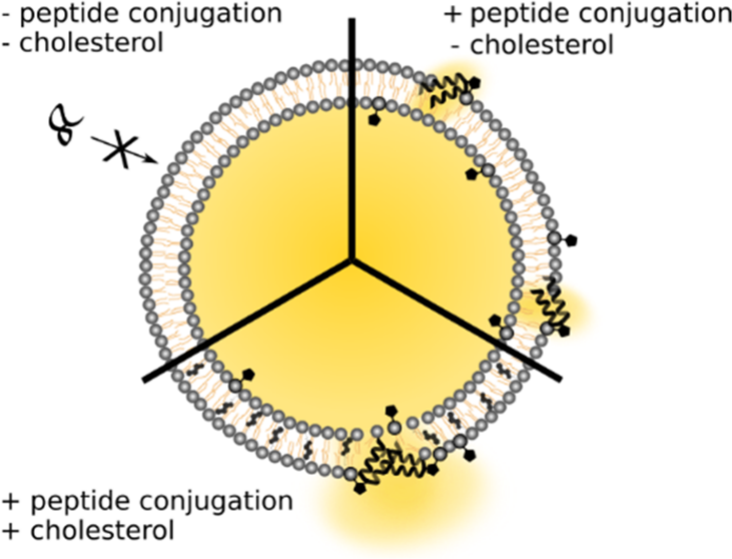

Liposome-based drug delivery systems are widely used to improve drug pharmacokinetics but can suffer from slow and unspecific release of encapsulated drugs. Membrane-active peptides, based on sequences derived or inspired from antimicrobial peptides (AMPs), could offer means to trigger and control the release. Cholesterol is used in most liposomal drug delivery systems (DDS) to improve the stability of the formulation, but the activity of AMPs on cholesterol-rich membranes tends to be very low, complicating peptide-triggered release strategies. Here, we show a de novo designed AMP-mimetic peptide that efficiently triggers content release from cholesterol-containing lipid vesicles when covalently conjugated to headgroup-functionalized lipids. Binding to vesicles induces peptide folding and triggers a lipid phase separation, which in the presence of cholesterol results in high local peptide concentrations at the lipid bilayer surface and rapid content release. We anticipate that these results will facilitate the development of peptide-based strategies for controlling and triggering drug release from liposomal drug delivery systems.

## Introduction

Liposomes are widely used drug delivery systems.^1^ Drug molecules can be efficiently encapsulated in liposomes and when combined with techniques for obtaining stable liposomes with long circulation times, significant improvements in drug pharmacokinetics and biodistribution can be achieved. Around 10 liposome-based drug formulations are in clinical use for the treatment of, e.g., breast cancer, ovarian cancer, leukemia, and fungal infections, and several liposome-based drug formulations are in various stages of clinical trials.^2^ However, for the liposome-encapsulated drugs to become bioavailable and provide a therapeutic effect, they must be released, which typically is a slow process that relies on passive diffusion and liposomal degradation.^2^ Membrane-active peptides can promote the release process by triggering lipid membrane disruption. Several strategies to use membrane-active peptides for controlled liposomal content release have been proposed,^3^ including possibilities to use peptide liposome conjugation for increased selectivity^4,5^ as well as means to control peptide membrane activity by proteases^6−8^ or phosphatases^7^ to allow for disease biomarker-controlled release. These peptides are usually based on sequences derived or inspired from antimicrobial peptides (AMPs).

AMPs are short, unstructured, and often cationic amphipathic membrane-active peptides that are very diverse with respect to both primary sequence and function. AMPs primarily interact with lipid membranes through unspecific electrostatic and hydrophobic interactions^9^ resulting in destabilization of lipid membrane integrity and loss in membrane potential, which can result in killing of bacteria. AMPs that are effective against both Gram-positive and Gram-negative bacteria,^10^ including antibiotic-resistant bacteria,^11^ have been identified. The membrane activity of AMPs is typically not very membrane-selective and dependent on both intrinsic factors relating to the properties of the peptides^12^ and the physiochemical properties of the lipid membranes.^13,14^ Several different models have been proposed to describe AMP-lipid membrane interactions,^12^ including the formation of barrel-stave or toroidal pores,^15,16^ or by carpet or detergent-like models.^17^ Some AMPs have also been observed to induce a separation of lipid components, resulting in clustering of anionic lipids and potentially formation of phase boundary defects between lipid domains.^18−20^ Such induced lateral phase separation could potentially contribute to the antimicrobial activity of AMPs.

Cholesterol is a major component of the eukaryotic cell membrane and has a large influence on lipid membrane fluidity,^21^ lipid packing,^22,23^ and membrane permeability.^24^ Cholesterol is also a major component in many liposomal drug delivery systems to increase liposomal stability and prevent premature drug release.^1^ Bacterial membranes lack cholesterol, which in combination with the presence of more anionic lipids render them more susceptible to AMPs compared to eukaryotic cells^25,26^ and other cholesterol-containing lipid membranes.^27^ However, in multicomponent bilayer mixtures, cholesterol can promote the formation of liquid-ordered (*L*_o_) lipid domains resulting in phase separation.^28^ Noticeably, although high cholesterol content is thought to limit AMP activity on cholesterol-rich membranes,^23^ it has been shown that AMP activity in heterogeneous lipid phase-separated model membranes is greatly enhanced compared to that in homogeneous membranes without abilities to phase-separate.^29,30^ Thus, lateral phase separation in cholesterol-containing membranes can also promote the formation of lipid phases with more favorable peptide interactions as opposed to homogeneous bilayers. It has also been observed that lipid line tension at heterogeneous membrane domain boundaries is an important factor that enhances the efficiency of viral peptide-mediated membrane fusion.^31^ HIV pseudoviruses appear to fuse with model membranes containing micron-sized coexisting *L*_o_- and liquid-disordered (*L*_d_)-phase domains at the edges rather than in the central areas of *L*_o_ domains. Peptide–membrane interactions are affected not only by preexisting lateral lipid phase domains but also by, e.g., antibody-mediated lipid cross-linking, which can result in a redistribution of lipids between phases.^32^ These processes are highly dependent on lipid acyl chain unsaturation and cholesterol content.^33^ There is consequently a complex relationship between peptide–membrane interactions and lipid phase domain formation, where the former can affect the latter and vice versa.

We have in recent work demonstrated that it is possible to design, synthesize, and optimize AMP-mimetic membrane-active peptides that selectively disrupt lipid membrane integrity of non-cholesterol-containing lipid bilayers when conjugated to headgroup-functionalized lipids.^4−6^ In addition, we have shown possibilities to rationally modulate and turn on the membrane activity of these peptides by means of very specific molecular interactions, including peptide heterodimerization and proteolytic cleavage of complementary peptides.^4,6^ Like many AMPs, these designed membrane-active peptides are cationic and amphipathic and modulate lipid membrane permeability through partition-folding coupling. The low affinity to lipid membranes, unless bound to specific lipids, can limit hemolytic and cytotoxicity effects, which combined with the numerous possibilities to control membrane activity and release of liposomal content, make these peptides highly interesting for the development of targeting strategies for AMPs and triggered release of liposomal drug delivery systems (DDS). However, since cholesterol is included in most liposomal DDS to enhance stability and prevent premature leakage of encapsulated drugs, possibilities to use AMP-derived peptides for modulating membrane integrity are limited.^34^

**Figure 1:**
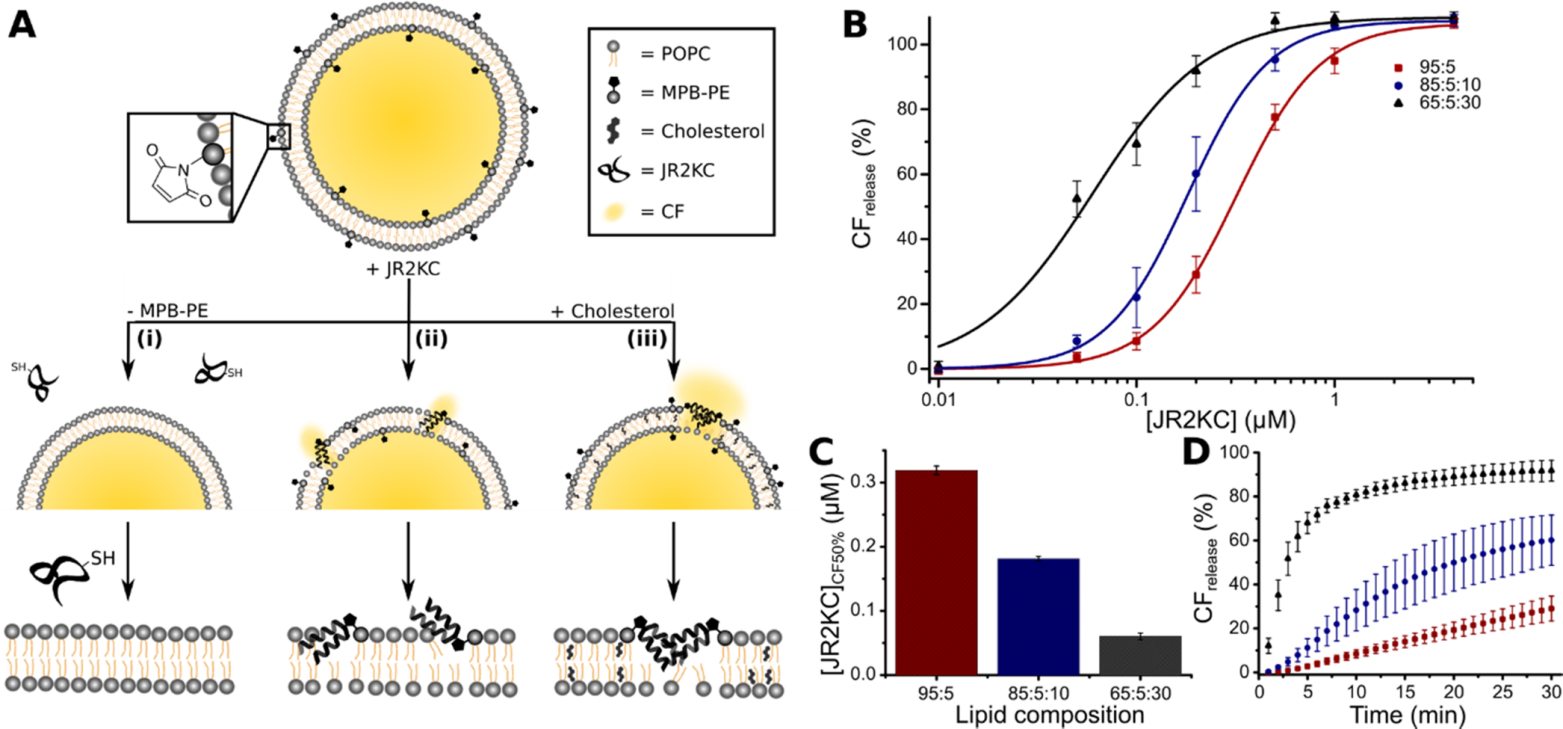
(A) Schematic illustration of peptide-mediated release of encapsulated CF. (i) Omitting MPB-PE prevents peptide coupling and subsequent CF release. (ii) JR2KC binds covalently to LUVs with MPB-PE, resulting in peptide folding and disruption of lipid membrane integrity, causing the release of encapsulated CF. (iii) Including cholesterol in the lipid bilayer results in peptide-induced lipid phase separation, resulting in high local peptide concentrations leading to a more rapid and efficient CF release. (B) Total CF release after 30 min incubation triggered by JR2KC (0.01–4 μM) on LUVs with 5 mol % MPB-PE and 0, 10, and 30 mol % cholesterol. Data were fitted to a Hill equation, *n* = 4. (C) The JR2KC concentration required to achieve 50% CF release ([JR2KC]_CF50%_) after 30 min incubation estimated from the fitting in (B). (D) CF release kinetics profile of CF from LUVs with 5 mol % MPB-PE and 0, 10, and 30 mol % cholesterol (red, blue, and black points, respectively) during 30 min incubation with 0.2 μM JR2KC. Error bars indicate standard deviation.

Here, we show a novel strategy to efficiently trigger release from cholesterol-rich lipid vesicles using designed membrane-active peptides and further explore in detail the molecular mechanisms involved (Figure 1A). We utilized a de novo designed membrane-active peptide (JR2KC)^6^ that previously has been demonstrated to trigger liposome destabilization when conjugated to headgroup-functionalized lipids. JR2KC is a 42-amino-acid polypeptide with a distinct positive net charge at neutral pH. As a monomer in solution, JR2KC exists as a random coil, but covalent conjugation to lipid bilayers via a cysteine (Cys) residue in the loop region to lipids with a maleimide headgroup has been shown to trigger folding into a well-defined α-helix.^6^ In vesicles, this process disrupts the integrity of the lipid membrane, resulting in content release. In contrast to AMPs, which are typically not active on homogeneous cholesterol-rich lipid membranes,^27^ we show a significant enhancement in JR2KC membrane activity when conjugated to large unilamellar vesicles (LUVs) with cholesterol compared to non-cholesterol-containing LUVs. Release of encapsulated carboxyfluorescein (CF) occurred more rapidly and at lower JR2KC concentrations in LUVs with cholesterol. The addition of JR2KC to homogeneous cholesterol-containing giant unilamellar vesicles (GUVs) resulted in the formation of distinct phase-separated domains, leading to local enrichment of JR2KC. This peptide-induced lipid restructuring, resulting in phase separation in cholesterol-containing lipid bilayers, was further confirmed by small- and wide-angle X-ray scattering (SAXS and WAXS). In addition, no CF release from any of the LUVs was seen upon the addition of a peptide with the identical primary sequence as JR2KC but where all l-alanines (l-Ala) were exchanged for d-ala, which prevents proper folding of the peptide. Conjugation of JR2KC to cholesterol-rich lipid membranes thus appears to lead to the formation of domains with a substantial increase in the local peptide concentration, promoting cooperative and peptide folding-dependent peptide–lipid membrane interactions, resulting in rapid and efficient membrane lysis.

In previous work, we have shown that we can inhibit the membrane activity of JR2KC by means of specific folding interactions with a complementary peptide (JR2E) followed by reactivation by proteolytic cleavage of the inhibiting peptide by a matrix metalloproteinase (MMP-7) overexpressed in tumors.^6^ The possibility to trigger the highly efficient release of encapsulated compounds from cholesterol-rich vesicles using selective peptide interactions, as demonstrated here, can further facilitate the development of advanced drug delivery systems, which we envision can provide new means for controlling and triggering drug release.

## Results and Discussion

### Influence of Cholesterol on Peptide-Triggered Release

To investigate the effect of cholesterol on vesicle stability in the presence of JR2KC, LUVs with varying amounts of cholesterol (Ch), 1-palmitoyl-2-oleoyl-*sn*-glycero-3-phosphocholine (POPC) and the maleimide headgroup-functionalized lipid 1,2-dioleoyl-*sn*-glycero-3-phosphoethanolamine-*N*-[4-(*p*-maleimidophenyl)butyramide] (MPB-PE) were prepared. As expected, including cholesterol resulted in reduced membrane fluidity and more ordered acyl chains with respect to POPC vesicles containing no cholesterol, as indicated by the increase in general polarization (GP) values of Laurdan,^35^ shifting from 0.069 in LUVs without cholesterol to 0.5 in LUVs with 30 mol % cholesterol (Figure S1). The release of carboxyfluorescein (CF), encapsulated in the LUVs at self-quenching concentrations (50 mM), was monitored over time when exposed to JR2KC in the concentration range of 0.01–4 μM. Prior to the addition of JR2KC, there was no, or very limited, CF release from any of the LUV compositions used (Figure S2A). The addition of JR2KC to LUVs with POPC/MPB-PE (95:5), i.e., without cholesterol, resulted in a distinct CF release for JR2KC concentrations ≥0.2 μM, as previously reported.^6^ Interestingly, when including cholesterol in the LUVs, a drastic increase in the release rate and extent was observed (Figures 1B and S3). The JR2KC concentration required to achieve 50% CF release in 30 min ([JR2KC]_CF50%_) was estimated by fitting the CF release profile to a Hill equation. In LUVs with 10 mol % cholesterol, i.e., POPC/MPB-PE/Ch (85:5:10), [JR2KC]_CF50%_ decreased from 0.32 to 0.18 μM compared to LUVs without cholesterol. When increasing the cholesterol content further to 30 mol % using LUVs with POPC/MPB-PE/Ch (65:5:30), [JR2KC]_CF50%_ was about 5.3 times lower compared to LUVs without cholesterol (Figure 1C). In addition to requiring lower peptide concentrations to trigger CF release, the CF release rate increased substantially with increasing cholesterol content (Figure 1D). At a JR2KC concentration of 0.2 μM, 50% CF release was reached within 20 and 3 min for LUVs with 10 and 30 mol % cholesterol, respectively, while LUVs without cholesterol did not reach above 35% CF release even after 30 min incubation at 5 mol % MPB-PE. In contrast to AMPs, the membrane lytic activity of JR2KC thus appears to be more pronounced on cholesterol-containing lipid bilayers. However, the peptide must still be conjugated to trigger any CF release and no release was seen from vesicles lacking MPB-PE (Figure S2B) or when using a peptide without the Cys residue in the loop region.^6^

**Figure 2:**
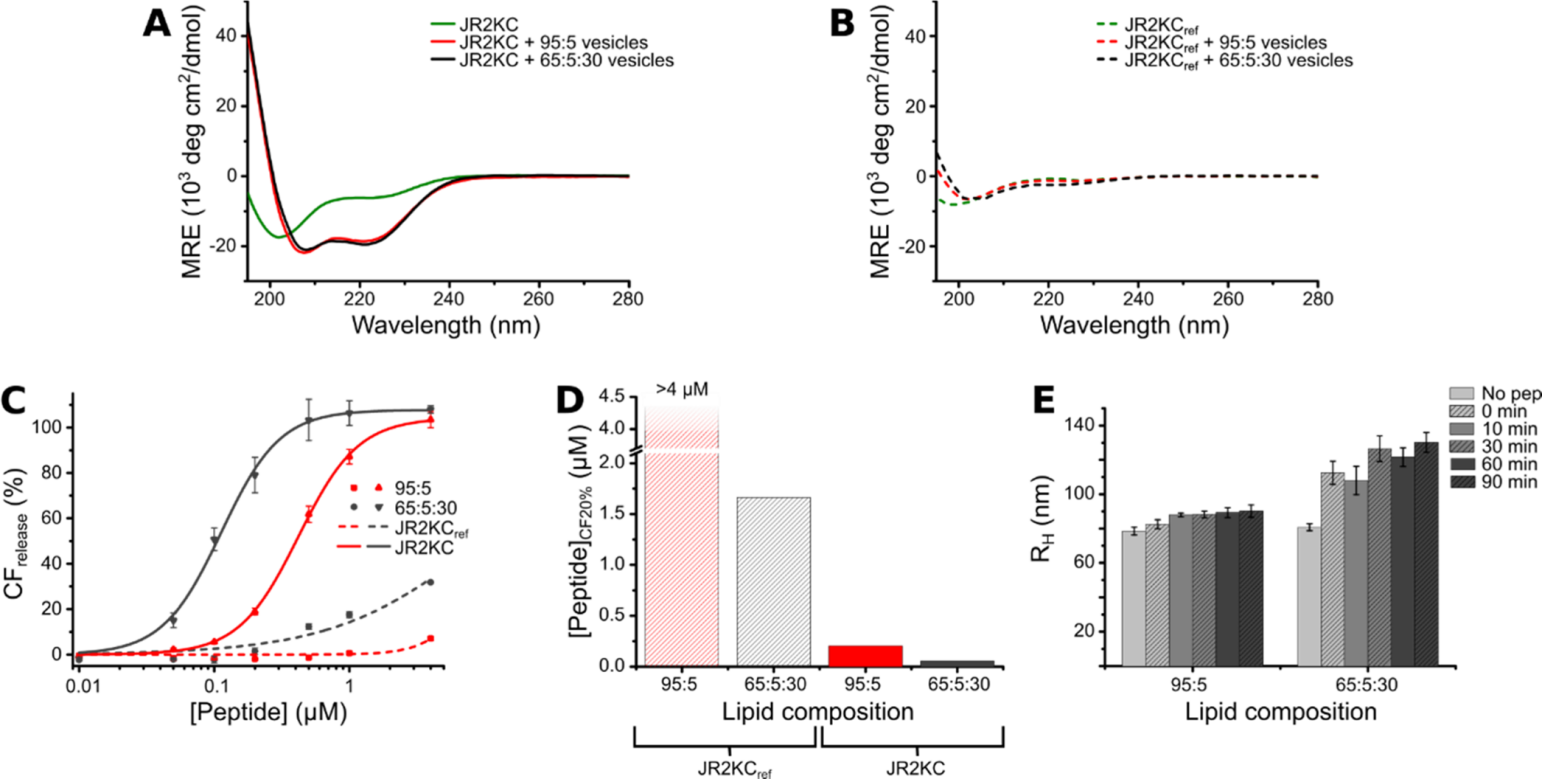
CD spectra of 30 μM (A) JR2KC and (B) JR2KC_ref_ in 0.01 M PB; alone (green), with LUVs with POPC/MPB-PE (95:5) (red) and POPC/MPB-PE/Ch (65:5:30) (black), lipid concentration 1.2 mM. The peptide:maleimide ratio was 1:2, and the peptides were incubated with the vesicles for >30 min before measurements. (C) Total CF release after 30 min using JR2KC (solid) and JR2KC_ref_ (dashed) on POPC/MPB-PE (95:5) vesicles (red) or POPC/MPB-PE/Ch (65:5:30) vesicles (black). Release curves were fitted to a Hill equation, *n* = 2. Error bars indicate standard deviation. (D) The peptide concentration needed to achieve 20% CF release within 30 min, estimated from the fitting in (C). (E) Changes in hydrodynamic radius (*R*_H_) of 40 μM POPC/MPB-PE (95:5) and POPC/MPB-PE/Ch (65:5:30) over time after incubation with 4 μM JR2KC, corresponding to a peptide:maleimide ratio of 2:1. Error bars represent the relative peak width.

JR2KC exists as a random coil in solution at neutral pH, but conjugation to lipid membranes induces folding into a well-defined α-helical structure (Figure 2A), which correlates well with our previous observations that the membrane activity of JR2KC is folding-dependent.^6^ The helicity was very similar for JR2KC conjugated to both cholesterol- and non-cholesterol-containing LUVs and comparable to the fully folded peptide in solution at pH > 8.^36^ To further confirm the importance of folding on the membrane activity of JR2KC in LUVs with cholesterol, we synthesized a peptide with the identical sequence to JR2KC but where all alanines (Ala) were replaced with the d-isomer. Since the incorporation of d-amino acids has a destabilizing effect on the amphipathic α-helical structure,^37^ this peptide (JR2KC_ref_) is unable to fold and adopt a defined secondary structure. As expected, JR2KC_ref_ did not fold in the presence of POPC/MPB-PE (95:5) vesicles nor with vesicles containing cholesterol (POPC/MPB-PE/Ch (65:5:30)) (Figure 2B). This correlates with the CF release data where essentially no release was observed when exposing the LUVs without cholesterol to JR2KC_ref_ (Figure 2C). A small amount of CF release (<35%) was seen in cholesterol-containing LUVs after 30 min incubation with the highest JR2KC_ref_ concentration (4 μM), but significantly lower than for JR2KC, and presumably caused by unspecific interactions mediated by the high local surface concentration of the positively charged peptides. As a comparison, the concentration of JR2KC_ref_ required to achieve 20% CF release during 30 min incubation with POPC/MPB-PE/Ch (65:5:30) LUVs was 30 times higher than for JR2KC (Figure 2D).

After establishing the coupling between peptide folding and perturbation of membrane integrity, we set out to understand the role of cholesterol in this process. Dynamic light scattering (DLS) was used to investigate the effect of JR2KC on the stability of the LUVs. A minor increase in hydrodynamic radius (*R*_H_) over time was observed after the addition of JR2KC to vesicles without cholesterol (Figure 2E and SI Figure S4). For vesicles with 30 mol % cholesterol, the increase in size was more pronounced, but since no major aggregation or micellization was observed for any of the LUV compositions, a carpet and/or detergent mechanism, resulting in lipid bilayer disintegration, can thus be ruled out. The increase in *R*_H_ of the cholesterol-containing vesicles indicates extensive peptide accumulation at the vesicle surface. Peptide insertion in the lipid bilayer can result in swelling and potentially reduce the colloidal stability of the vesicles.

**Figure 3:**
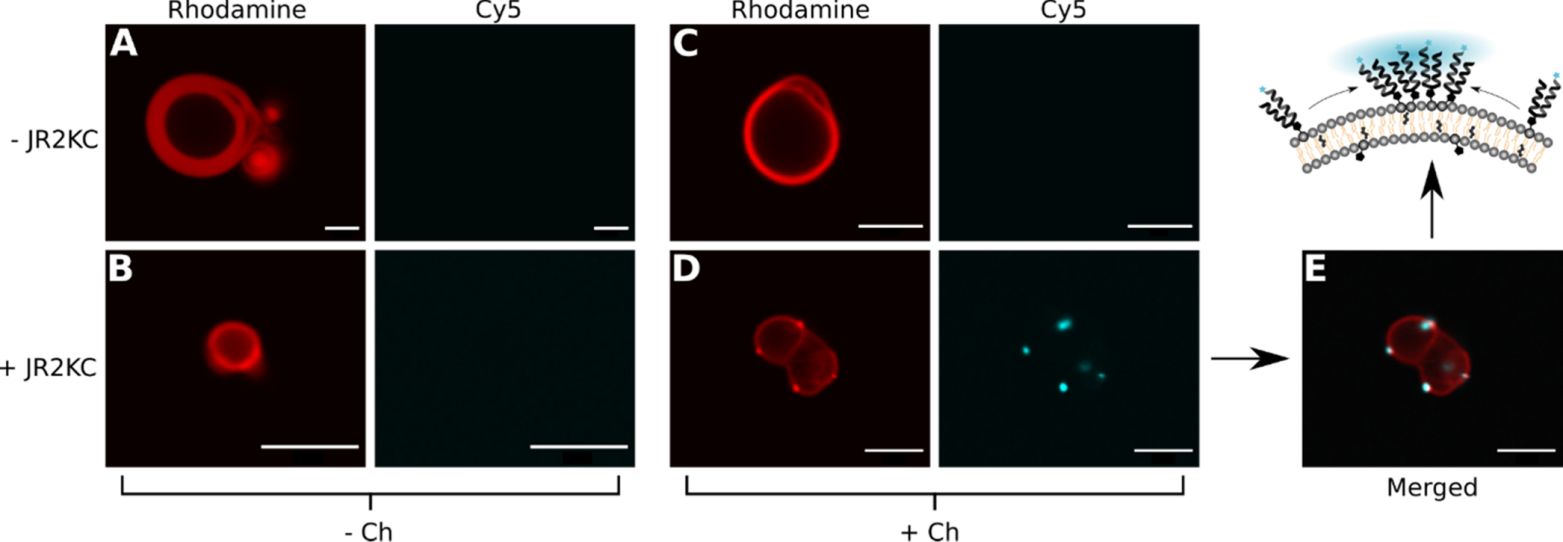
Confocal fluorescence microscopy images of GUVs composed of (A, B) 95:5:0.5 POPC/MPB-PE/Liss Rhod-PE and (C, D) 65:5:30:0.5 POPC/MPB-PE/Ch/Liss Rhod-PE. Columns as indicated show rhodamine fluorescence (λ_ex_ = 561 nm and λ_em_ = 570–600 nm) and Cy5 fluorescence (λ_ex_ = 633 nm and λ_em_ = 650–690 nm). (A, C) GUVs without JR2KC-Cy5 addition; (B, D) GUVs with JR2KC-Cy5 addition. (E) Merged picture of the rhodamine and Cy5 images shown in (D). Scale bars: 5 μm.

Since cholesterol can induce the formation of lipid phase domains with distinctly different lipid composition and order,^38^ we hypothesized that phase separation could contribute to the peptide accumulation process and the efficient release from cholesterol-containing vesicles. To investigate this hypothesis further, we prepared giant unilamellar vesicles (GUVs) containing 0.5 mol % of the rhodamine-labeled lipid 1,2-dioleoyl-*sn*-glycero-3-phosphoethanolamine-*N*-(lissamine rhodamine B sulfonyl) (Liss Rhod-PE), in combination with either POPC/MPB-PE (95:5) or POPC/MPB-PE/Ch (65:5:30), to facilitate imaging of the vesicles using confocal fluorescence microscopy. Liss Rhod-PE has an identical acyl chain composition to MPB-PE, which should make these two lipids assemble in the same lipid phase in case of lipid phase separation. In vesicles without cholesterol, rhodamine fluorescence was homogeneously distributed in the lipid bilayer and no phase inhomogeneities could be observed, neither before (Figure 3A) nor after (Figure 3B) the addition of JR2KC. Vesicles with cholesterol also displayed a homogeneous distribution of the rhodamine-labeled lipids but only before the addition of JR2KC (Figure 3C). Upon the addition of JR2KC, distinct bright spots were observed in the membrane of the GUVs, clearly indicating phase separation (Figure 3D). To investigate in which phase the peptides were localized, JR2KC was labeled with the fluorescent dye Cy5. When JR2KC-Cy5 was introduced to GUVs lacking cholesterol (Figure 3B), no fluorescence was seen in the lipid membrane although both CD spectroscopy and CF release data clearly show that the peptides are bound to the lipid bilayer. This indicates that the peptides are homogeneously distributed in the lipid bilayer, resulting in a diluted fluorescence signal. In GUVs with 30% cholesterol, however, distinct JR2KC-Cy5 fluorescence was observed, which was co-localized with the brighter rhodamine domains (Figures 3D,E and S5). From these observations, we can thus conclude that conjugation of JR2KC to the vesicles triggers a folding-dependent phase separation. This cholesterol-dependent and peptide-mediated lipid rearrangement results in the formation of distinct phase domains. In addition to contributing to a high local peptide concentration, likely facilitating cooperative peptide–peptide interactions, the induced line tension between the formed lipid phase boundaries could potentially be more susceptible to peptide insertion resulting in a more pronounced membrane destabilization, driven by the entropic gain upon peptide folding and partitioning in the lipid bilayer.

### Influence of Lipid Properties on JR2KC-Triggered Release from Cholesterol-Containing Vesicles

**Figure 4:**
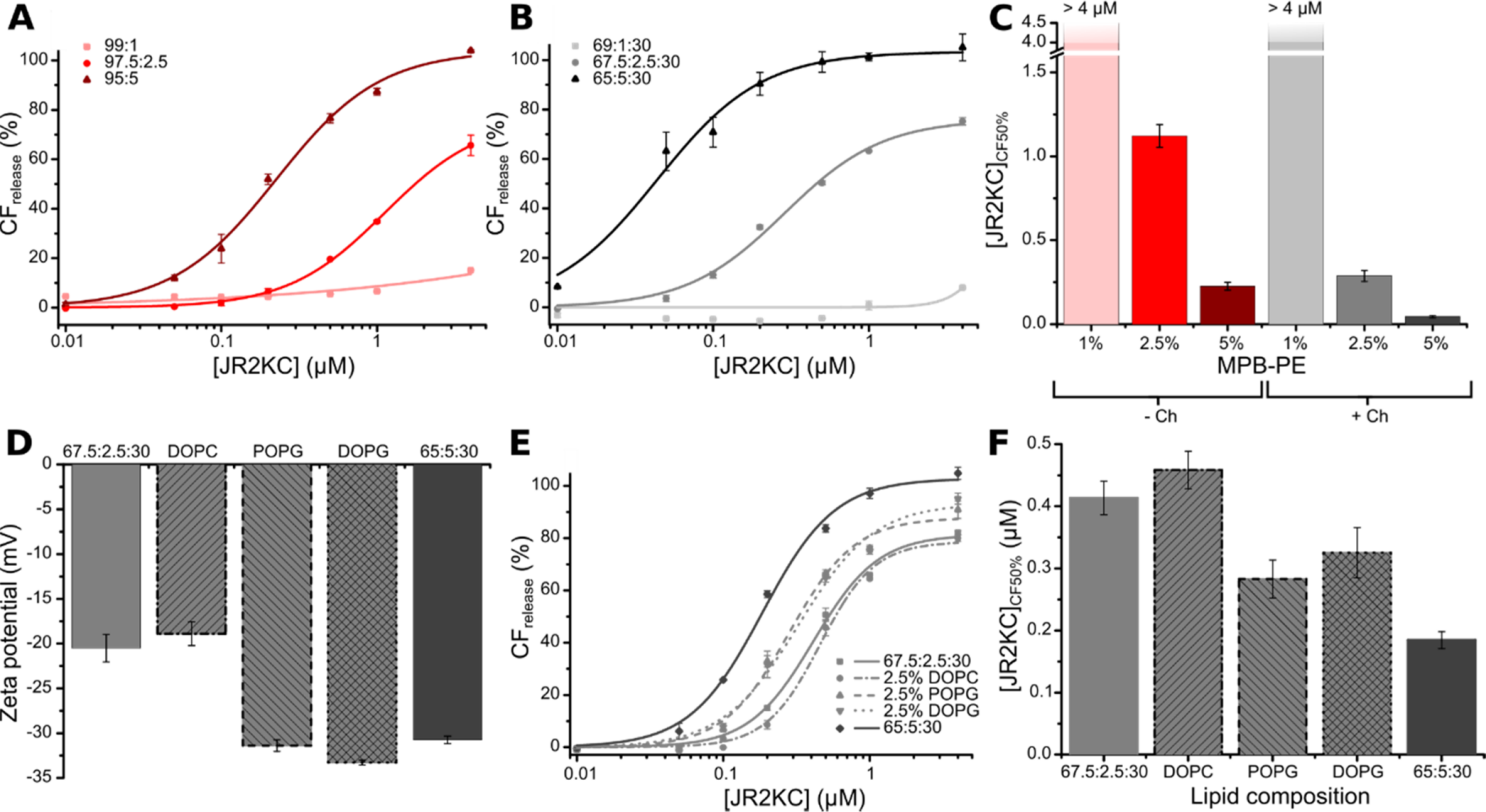
(A, B) Total CF release after 30 min incubation with JR2KC using 1, 2.5, and 5 mol % MPB-PE in (A) POPC/MPB-PE vesicles and (B) POPC/MPB-PE/Ch vesicles (fixed 30 mol % cholesterol), fitted to a Hill equation, *n* = 2. (C) The JR2KC concentration needed to achieve 50% CF release after 30 min obtained from the fittings in (A) and (B). (D) ζ potential of vesicles used in (E) and (F). (E) Total CF release after 30 min incubation with JR2KC from vesicles with POPC/MPB-PE/Ch (67.5:2.5:30 and 65:5:30) and POPC/MPB-PE/Ch/DOPC, POPC/MPB-PE/Ch/POPG, and POPC/MPB-PE/Ch/DOPG (65:2.5:30:2.5). Data were fitted to a Hill equation, *n* = 2. (F) [JR2KC]_CF50%_ obatined from the data and LUV compositions as indicated in E. Error bars are standard deviation.

We have previously seen that JR2KC-triggered release is highly dependent on the mol % MPB-PE.^6^ Here, a drastic decrease in CF release was observed for LUVs both with and without cholesterol when the concentration of MPB-PE was reduced from 5 to 1 mol %, even for the highest peptide concentrations used (Figure 4A,B). At a concentration of 2.5 mol % MPB-PE, the release was still substantially lower than at 5 mol % but clearly higher in vesicles containing cholesterol. [JR2KC]_CF50%_ was about a factor 4 lower in cholesterol-containing vesicles compared to vesicles without cholesterol (Figure 4C) and comparable to [JR2KC]_CF50%_ for vesicles with 5 mol % MPB-PE without cholesterol. The presence of cholesterol can consequently compensate for a lower concentration of MPB-PE and a lower concentration of anchored peptides. Thus, although the total surface concentration of JR2KC bound to the vesicles appears to be the limiting factor for efficient release, the presence of cholesterol drastically improves the release kinetics. Other factors may, however, contribute to this process, such as lipid net charge and acyl chain composition. To systematically investigate the effects of altering these parameters, a series of vesicles with lipid compositions comprising 65 mol % POPC, 2.5 mol % MPB-PE, 30 mol % cholesterol, and 2.5 mol % of a fourth lipid species (DOPC/POPG/DOPG) were prepared. In addition to reducing the number of binding sites for JR2KC, reduction of the amount of MPB-PE also results in a lower vesicle net charge. Since JR2KC has a net charge of +11 at pH 7, fewer negatively charged lipids could reduce the electrostatic interactions between the peptides and the vesicle surface resulting in both a lower rate of surface accumulation and less favorable interactions. Moreover, considering that the difference in acyl chain saturation between POPC and MPB-PE is likely contributing to phase separation, incorporation of a fourth lipid species, carrying the same acyl chains as MPB-PE (1,2-dioleoyl), could compensate for a decrease in MPB-PE concentration and contribute to phase separation. Thus, we prepared vesicles with the following compositions: POPC/MPB-PE/Ch/DOPC, POPC/MPB-PE/Ch/POPG, and POPC/MPB-PE/Ch/DOPG (65:2.5:30:2.5) and compared the JR2KC-triggered release with vesicles comprising POPC/MPB-PE/Ch (67.5:2.5:30) and (65:5:30). DOPC has the same acyl chains as MPB-PE but shares the same zwitterionic headgroup as POPC. POPG on the other hand shares the same acyl chains as POPC but with a negatively charged headgroup, like MPB-PE. DOPG has both the same acyl chains and negative charge on the headgroup as MPB-PE. The properties of the lipids are summarized in Table 1. ζ potential measurements of the different vesicles show net charges as expected where vesicles with 5 mol % of charged lipids (65:5:30 POPC/MPB/Ch and 67.5:2.5:30:2.5 POPC/MPB/Ch/POPG or DOPG) displayed ζ potentials around −30 mV, while vesicles comprising 2.5 mol % charged lipids (67.5:2.5:30 POPC/MPB/Ch and 67.5:2.5:30:2.5 POPC/MPB/Ch/DOPC) were slightly less negative with ζ potentials of about −20 mV (Figure 4D). The CF release after the addition of JR2KC clearly shows that the addition of 2.5 mol % DOPC did not have any significant effect on the CF release, and [JR2KC]_CF50%_ was comparable to vesicles with 2.5 mol % MPB-PE (Figure 4E,F). On the other hand, POPG and DOPG did improve the release kinetics slightly, indicating that the overall vesicle charge is a more important parameter for the peptide-mediated release than the presence of unsaturated acyl chains. None of the combinations resulted in a release that was as rapid and efficient as for vesicles containing 5 mol % MPB-PE, showing that, in addition to the phase separation mediated by cholesterol, both lipid net charge and peptide surface concentration are critical factors for efficient release.

**Table 1:** Summary of Lipid Properties

	POPC	MPB-PE	DOPC	POPG	DOPG
net charge	±	–	±	–	–
lipid headgroup	phosphocholine	phosphoethanolamine-*N*-[4-(*p*-maleimidophenyl)butyramide]	phosphocholine	phospho-(1′-*rac*-glycerol)	phospho-(1′-*rac*-glycerol)
acyl chains	16:0/18:1	18:1/18:1	18:1/18:1	16:0/18:1	18:1/18:1
*T*_m_ (°C)	–2	N.D.	–17	–2	–18

### Influence of Cholesterol, MPB-PE, and JR2KC on POPC-Based Lipid Membrane Structures

**Figure 5:**
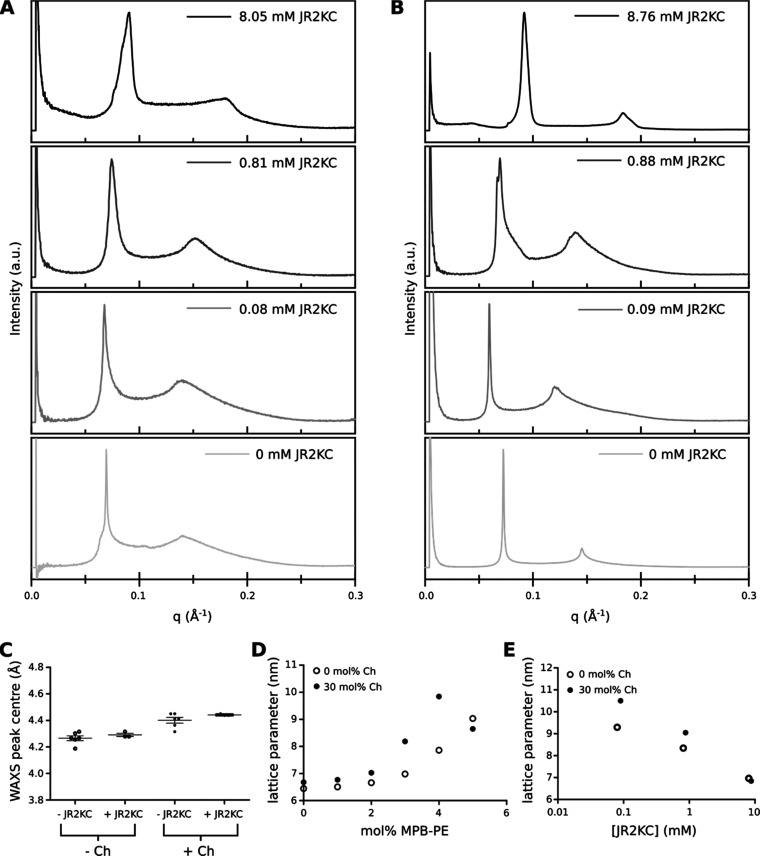
Summary of SAXS/WAXS data obtained at room temperature. (A, B) SAXS data for the addition of peptide at three concentrations to lipid mixtures containing POPC with 5 mol % MPB-PE and (A) 0 mol % cholesterol (B) 30 mol % cholesterol. (C) Position of WAXS peak maximum for all measured lipid compositions. (D) Effect of MPB-PE: SAXS data fitting for lipid mixtures with 0 and 30 mol % cholesterol (no peptide). (E) Effect of peptide: SAXS data fitting for lipid mixtures with 5 mol % MPB-PE (with peptide). For C, D, E each data point represents an individual measurement. In C, the error bars represent the mean and standard deviation of *n* = 3 (+JR2KC) or *n* = 5 (−JR2KC).

DLS data showed that JR2KC-mediated release of CF from vesicles did not induce rupture or significantly influence vesicle stability. Therefore, changes in the lipid membrane structure could play a role in the CF leakage measured from vesicles with incorporated MPB-PE and cholesterol. To study this, we have used SAXS/WAXS to characterize bulk lipid mixtures mimicking the vesicle release assay conditions to investigate structural changes to the POPC lipid membrane as a result of cholesterol content (0, 30 mol %), MPB-PE content (0–5 mol %), and using the same peptide:MPB-PE ratio (1:2, 1:20 and 1:200) as in the CF release experiments (Figure 5A,B).

At 0 mol % MPB-PE, the POPC lipid mixtures (0, 30 mol % cholesterol) are homogeneous and exhibit a single lamellar phase in SAXS scattering patterns, i.e., stacked bilayer phase morphology (see Figure S6A,B). Lack of peaks corresponding to cholesterol crystals in the SAXS indicates that the 30 mol % cholesterol is fully incorporated into the lipid bilayer. The WAXS data show a broad peak centered at 4.26 ± 0.04 Å (0 mol % cholesterol) or 4.40 ± 0.05 Å (30 mol % cholesterol) indicating a disordered and fluid-like packing of the lipid hydrocarbon chains (Figure 5C).^39^ The small shift in the fluid WAXS peak upon addition of cholesterol is due to an increase in the average separation between phospholipid/cholesterol molecules.^40^ For binary mixtures of POPC (70 mol %) with cholesterol (30 mol %), some phase diagrams have reported a *L*_o_–*L*_d_ phase coexistence.^41,42^ The single lamellar phase detected at 30 mol % cholesterol implies either that the lattice parameters of the *L*_o_ and *L*_d_ phases are identical or that the percentage of *L*_d_ phase is below the limit of detection as reported previously.^43^

Dye release studies demonstrated that the CF dye release was most efficient at 5 mol % MPB-PE and 30 mol % cholesterol. Incorporating the negatively charged lipid MPB-PE (0–5 mol %) into the lipid membrane induces a net increase in the lattice parameter (here, the sum of the bilayer thickness and coordinated water later), most likely due to an increase in the coordinated water layer with an increased negative charge (Figure 5D). We also observed that adding 30 mol % cholesterol to POPC containing 5 mol % MPB-PE increases the lattice parameter by 0.23 ± 0.00 nm. This agrees well with previous observations that the addition of 30 mol % cholesterol to a POPC lipid membrane thickens the lipid bilayer.^43^ In the absence of JR2KC, MPB-PE-containing POPC samples with 30 mol % cholesterol were homogeneous, indicating uniform MPB-PE incorporation (Figure S6B). However, MPB-PB-containing POPC samples with no cholesterol exhibited a broader range of lattice parameters, indicative of additional disorder to the lamellar phase behavior in POPC and potentially MPB-PE clustering (Figure S6A). Upon MPB-PE incorporation, no change was observed in the packing of the hydrocarbon chains in WAXS scattering patterns in both the cholesterol-containing and pure POPC samples (Figure S6, insets), indicating that these lipid mixtures still exhibit fluid phase behavior.

At the highest JR2KC concentration, the lattice parameter of both the 0 and 30 mol % cholesterol lipid mixtures decreased to a similar size to those observed with 0 mol % MPB-PE (Figure 5D,E). The covalent maleimide–cysteine coupling between MPB-PE and JR2KC anchors the peptide to the lipid membrane surface, potentially facilitating electrostatic interactions between the negatively charged lipid and the positively charged peptide. The result of this interaction is a reduction of the coordinated water layer and therefore decreased lattice parameter. At increasing peptide concentrations, we also observed peak broadening in the SAXS data caused by the presence of a range of lattice parameters, which indicates peptide-induced phase separation. This phase separation could be a result of *L*_o_–*L*_d_ phase coexistence (at 30 mol % cholesterol)^41,42^ or a clustering of the negatively charged lipid (MPB-PE) in the lipid membrane.^44^ The WAXS indicates a continued fluid state, i.e., no gel formation, in all of the samples measured, with no changes to the WAXS peak position upon addition of JR2KC (Figure 5C). This indicates that JR2KC does not impact the packing of the hydrocarbon chains. In the 30 mol % cholesterol-containing lipid membrane, previous reports of *L*_o_ and *L*_d_ phase coexistence with POPC with 30 mol % cholesterol suggest that the lipid mixture is either already phase-separated with a minimal proportion of *L*_d_ phase (as shown by the SAXS data) or close to a phase separation boundary. Therefore, the mixture may be more likely to phase-separate further upon the addition of JR2KC, which can induce electrostatic clustering of the MPB-PE. The observed JR2KC-mediated leakage is concentration-dependent; therefore, increased phase separation in a cholesterol-containing membrane either from *L*_o_ to *L*_d_ coexistence or clustering of MPB-PE could induce CF leakage at a faster rate. Previous reports have shown that while there are some permeability differences between different liquid phases and from gel phases, the highest leakage is at the domain boundaries or close to a phase transition.^45,46^ This phase separation is supported by the GUV data, which show that for 30 mol %, distinct regions of JR2KC are visible, compared to an even distribution in the 0 mol % cholesterol mixture. Therefore, a suggested mechanism of action for JR2KC-mediated leakage via the lipid membrane is via clustering of JR2KC potentially mediated by phase separation. This would lead to increased leakage in JR2KC rich regions compared to regions with lower concentrations.

## Conclusions

We have shown that the incorporation of cholesterol in POPC-based vesicles greatly influences their interaction with the AMP-mimetic peptide JR2KC. Dye release experiments using vesicles encapsulating self-quenching concentrations of CF showed that a significantly lower concentration of JR2KC was required to trigger release from cholesterol-containing vesicles compared to vesicles without cholesterol. We also observed a dramatic increase in release kinetics in the presence of cholesterol. This process required conjugation of JR2KC to the lipid membranes and was peptide folding-dependent. A peptide (JR2KC_ref_) with identical primary sequence to JR2KC but unable to fold due to a mix of amino acid isomers proved unable to induce release. The increased membrane activity of JR2KC in cholesterol-containing vesicles was not a consequence of vesicle aggregation but could instead be linked to lipid phase separation induced by the conjugation and folding of the peptides. The lipid membrane restructuring was confirmed by confocal fluorescence microscopy images of GUVs and SAXS/WAXS data, indicating that the increased membrane activity was enabled by higher local peptide concentrations originating from the induced lipid phase separation, possibly facilitated by electrostatic interactions between the positively charged peptide and the negatively charged headgroup-functionalized lipids. These observations demonstrate that a designed membrane-active peptide can trigger phase separation in POPC-based vesicles, which in the presence of cholesterol can efficiently trigger the release of vesicle content by means of very specific folding-dependent interactions. The possibility to modulate vesicle permeability by means of very specific and tunable interactions could provide an interesting route for the further development of advanced drug delivery systems that could circumvent the issues with passive drug release.

## Materials and Methods

### General

All lipids were purchased from Avanti Polar Lipids (Alabaster). All other chemicals were acquired from Sigma-Aldrich (Sigma-Aldrich, Saint Louis, Missouri).

### Peptide Synthesis

Fmoc based solid-phase peptide synthesis was used to obtain both JR2KC (H_2_N-NAADLKKAIKALKKHLKAKGPCDAAQLKKQLKQAFKAFKRAG-COOH) and JR2KC_ref_ (JR2KC with all l*-*Ala exchanged for d*-*Ala). JR2KC was synthesized as described earlier,^47^ whereas JR2KC_ref_ was synthesized on a Liberty Blue Automated Microwave Peptide Synthesizer (CEM, Matthews, North Carolina) using a Cl-MPA ProTide resin (CEM, 0.16 mmol/g) on a 100 μmol scale. The first amino acid was attached using a mixture of anhydrous KI and diisopropylethylamine (DIEA) in DMF under microwave conditions. All remaining couplings were performed twice using microwave conditions with a 5-fold excess of amino acid, Oxyma pure (CEM) as a base, and *N*,*N*′-diisopropylcarbodiimide (DIC) as a coupling reagent. The crude peptide was cleaved from its solid support by treatment with trifluoracetic acid (TFA)/H_2_O/triisopropylsilane (TIPS)/3,6-dioxa-1,8-octanedithiol (DODT)(92.5/2.5/2.5/2.5, v/v/v/v) followed by concentration and precipitation in cold diethyl ether. After synthesis, the crude peptide was purified using an RP-HPLC system (Dionex/Thermo Fisher, Waltham, Massachusetts) with an aquatic gradient of acetonitrile under acidic conditions, and the final product was verified with MALDI-ToF mass spectroscopy. Analytical RP-HPLC with an aquatic gradient of acetonitrile containing 0.1% TFA was used to confirm peptide purity (SI Figure S7). For confocal microscopy imaging, JR2KC was further labeled with sulfo-cyanine5 NHS ester (Cy5, Lumiprobe GmbH, Hannover, Germany) in a 1:1 molar ratio and incubated for 4 h at room temperature followed by gel filtration on a PD-10 column to remove uncoupled Cy5.

### Peptide Concentration

Since only reduced peptides with free thiols are capable of coupling to the lipid bilayer, the peptide concentration was set to the concentration of active thiols. This was determined using Ellman’s test^48^ prior to each experiment.

### LUV Preparation

Large unilamellar vesicles were prepared by thin-film hydration followed by extrusion. Lipids dissolved in chloroform were mixed at the desired molar ratios, and the chloroform was evaporated using a stream of nitrogen, creating a lipid film. To ensure solvent removal, the lipid film was placed in a vacuum desiccator overnight followed by rehydration with 0.01 M PBS (140 mM sodium chloride, 2.7 mM potassium chloride, and 10 mM phosphate), pH 7.4, or 0.01 M phosphate (PB), pH 7.4, for 10 min, and subsequently vortexed for 1 min, resulting in a total lipid concentration of 5 mg/mL. The lipid suspensions were extruded 21 times through a 100 nm polycarbonate membrane using a mini extruder (Avanti Polar Lipids, Alabaster, Alabama) to produce monodisperse vesicles. For Laurdan encapsulation, 0.3 mol % Laurdan dissolved in chloroform was added to the lipid mixture before solvent evaporation and then the procedure was as described above with the exception that everything was performed in the dark to limit photobleaching. CF-loaded vesicles were prepared as described above with the exception that 50 mM CF (self-quenching concentrations) dissolved in 10 mM PB with 90 mM NaCl (pH adjusted to 7.4) was used to rehydrate the lipid film. Unencapsulated CF was removed by size exclusion chromatography on a G-25 column (GE) in PBS prior to further analysis.

### GUV Preparation

Giant unilamellar vesicles were prepared with the gentle hydration method. Lipid stocks, all dissolved in chloroform, were mixed in the molar ratios 95:5:0.5 (POPC/MPB-PE/Liss Rhod-PE) and 65:5:30:0.5 (POPC/MPB-PE/Ch/Liss Rhod-PE) and spread on a poly(tetrafluoroethylene) (PTFE) pad to create a lipid film. After 2 h in a vacuum desiccator, the lipid film was prehydrated using water-saturated nitrogen for at least 30 min. Following this, the PTFE pad was placed in a beaker and covered with PBS (0.01 M, pH 7.4). The beaker was sealed with parafilm and incubated overnight at 37–45 °C. The following day, GUVs could be collected.

### Laurdan Generalized Polarizability

Laurdan fluorescence intensity was obtained with a fluorescence plate reader (Tecan Infinite M1000 Pro, Tecan Austria GmbH, Grödig/Salzburg, Austria) using an excitation wavelength of 350 nm and recording the emission intensities between 370 and 600 nm. A total of 200 μM lipids were used resulting in a Laurdan concentration of 0.6 μM. Intensity values of 435 and 500 nm were then extracted to calculate the generalized polarization (GP) values^35^

### Carboxyfluorescein Release Assay

A fluorescence plate reader (Tecan Infinite M1000 Pro, Tecan Austria GmbH, Grödig/Salzburg, Austria), λ_ex_ = 485 nm and λ_em_ = 520 nm, was used to study the carboxyfluorescein (CF) release over time from vesicles encapsulating self-quenching concentrations of CF (50 mM). Vesicles were prepared in a 96-well plate using PBS (0.01 M, pH 7.4) to achieve the desired concentration (40 μM) and the fluorescence was measured (*F*_0_). The peptide was then added so that the total volume was 200 μL and the increase in fluorescence was measured over time (*F*) to study its kinetic. When finished, Triton X-100 was added (1% v/v) and the fluorescence was measured after 15 min incubation (*F*_tot_). The CF release at a chosen time point was then calculated using .

The final peptide concentrations in all release experiments were 0, 0.01, 0.05, 0.1, 0.2, 0.5, 1, and 4 μM peptide.

### Dynamic Light Scattering (DLS)

DLS was carried out at 22 °C on an ALV/DLS/SLS-5022F system from ALV-GmbH (Langen, Germany) equipped with a 632.8 nm HeNE laser. PBS (0.01 M, pH 7.4) was filtered prior to usage with a 0.22 μm filter, and the temperature was controlled using a thermostat bath. The lipid (40 μM) and peptide (4 μM) concentrations were chosen to match those of the CF release assay. All correlation curves are the average of 10 consecutive 30 s runs, and the CONTIN 2DP routine implemented in the ALV software was used to calculate the particle size distribution.

### Circular Dichroism (CD) Spectroscopy

A Chirascan (Applied Photophysics, Leatherhead, United Kingdom) was used to record the CD measurements which were performed at room temperature using a 1 mm pathlength quartz cuvette. Scanning was performed between 195 and 280 nm with steps of 0.5 nm. Each sample was prepared in PB (0.01 M, pH 7.4), which was also used as background for pure peptide measurements. Vesicles in PB were used as background for measurements on peptide-coupled vesicles. Peptide and lipid concentrations used were 30 μM and 1.2 mM, respectively, corresponding to a peptide:malemide ratio of 1:2. Five spectra of each sample were recorded and averaged, except for background references where only three spectra were used, and Savitzky–Golay algorithm was used to smoothen the curves. Spectra of peptide with vesicles were recorded after >30 min incubation.

### Imaging of GUVs with Confocal Microscopy

A Leica TCS SP5 confocal microscope (Leica Microsystems, Wetzlar, Germany) was used to image GUVs, with or without JR2KC addition. To facilitate image acquisition, the GUVs (120 μM) were immobilized in a 0.5% w/v low-melting agarose gel prior to imaging. For those samples including both vesicles and peptide, JR2KC-Cy5 was added after gel formation. A 561 nm laser was used to illuminate the rhodamine fluorophore (λ_em_ = 570–600 nm), while a 633 nm laser was used for the Cy5 fluorophore (λ_em_ = 650–690 nm). All images were obtained using a 25× objective (0.95 numeric order, water-based) and processed using ImageJ.

### ζ Potential

The ζ potential of vesicles was measured in 0.01 M PB (pH 7.4) using a Malvern ZetaSizer Nano ZS90 (Malvern Panalytical, Malvern, Worcestershire, United Kingdom).

### Data Fitting

A Hill equation was used to fit all data from the total CF release experiments (Figures 1B, 2C and 4A,B,D), where *x* is the peptide concentration, *k* is a constant, and *n* is the Hill coefficient 

### SAXS/WAXS

SAXS/WAXS data was collected at Diamond Light Source on the I22 beamline.^49^ Samples were prepared by co-dissolving lipids in chloroform in the desired molar ratios to a total mass of 15 mg lipids per sample. The chloroform was evaporated with a stream of nitrogen, and the formed lipid films were placed in a vacuum desiccator for 4 h followed by the addition of 60 μL of PBS (0.01 M, pH 7.4) or JR2KC (1:2, 1:20, or 1:200 peptide:MPB-PE ratio) in PBS, corresponding to 80 wt %. The vials were sealed to prevent water loss during the subsequent freeze–thaw process. First, the samples were centrifuged for 10 min, shortly vortexed, and incubated for 15 min followed by 15 cycles of freeze–thawing. Finally, the samples were centrifuged for 3 min and then kept in the freezer until loaded into polycarbonate capillaries provided by the Diamond Light Source, sealed with araldite, loaded into a capillary holder, and shipped at −20 °C. Before data collection, the capillary holder was thawed to room temperature. Simultaneous SAXS/WAXS data were collected at room temperature, using a monochromatic beam (12.4 keV) and a camera length of 3 m. For each capillary, 100 sequential images of 100 ms each were collected using an automated setup. SAXS/WAXS data were analyzed using DAWN software^50,51^ and Axcess,^52^ a custom in-house software.

## Supporting Information Available

Laurdan emission spectra and generalized polarization (GP) values; CF release kinetics for LUVs with POPC/MPB-PE/Ch at the ratios (100:0:0), (95:5:0), (85:5:10), (65:5:30), and (70:0:30) vesicles in the absence of JR2KC; kinetics for CF release by JR2KC (0.01–4 μM) on LUVs with 5 mol % MPB-PE and 0, 10, and 30 mol % cholesterol; DLS correlation functions and size distributions of LUVs after the addition of JR2KC; additional confocal fluorescence microscopy images of GUVs composed of POPC/MPB-PE/Ch/Liss Rhod-PE (65:5:30:0.5) incubated with JR2KC-Cy5; SAXS and WAXS scattering patterns; and analytical HPLC chromatogram of JR2KC_ref_ (PDF)
