# Supplementary material for: Peptide-Folding Triggered Phase Separation and Lipid Membrane Destabilization in Cholesterol-Rich Lipid Vesicles
Source: Bioconjug Chem. 2022 Apr 1;33(4):736–46. doi: 10.1021/acs.bioconjchem.2c00115 (PMC9026255; doi:10.1021/acs.bioconjchem.2c00115)
Supplement: Supplementary file 1 — bc2c00115_si_001.pdf [file bc2c00115_si_001.pdf]

Supporting Information

Peptide-Folding Triggered Phase Separation and Lipid Membrane Destabilization in Cholesterol-Rich Lipid Vesicles

Johanna Utterström[1], Hanna M. G. Barriga[2], Margaret N. Holme[,2], Robert Selegård[1], Molly M. Stevens[*,2,3], Daniel Aili[*,1]*

[1]Laboratory of Molecular Materials, Division of Biophysics and Bioengineering, Department of Physics, Chemistry and Biology, SE-581 83 Linköping, Sweden

[2]Department of Medical Biochemistry and Biophysics, Karolinska Institutet, SE-171 77 Stockholm, Sweden

[3]Department of Materials, Department of Bioengineering and Institute of Biomedical Engineering Imperial College London, London, SW7 2AZ, U.K.

*Corresponding authors: daniel.aili@liu.se, m.stevens@imperial.ac.uk, margaret.holme@ki.se

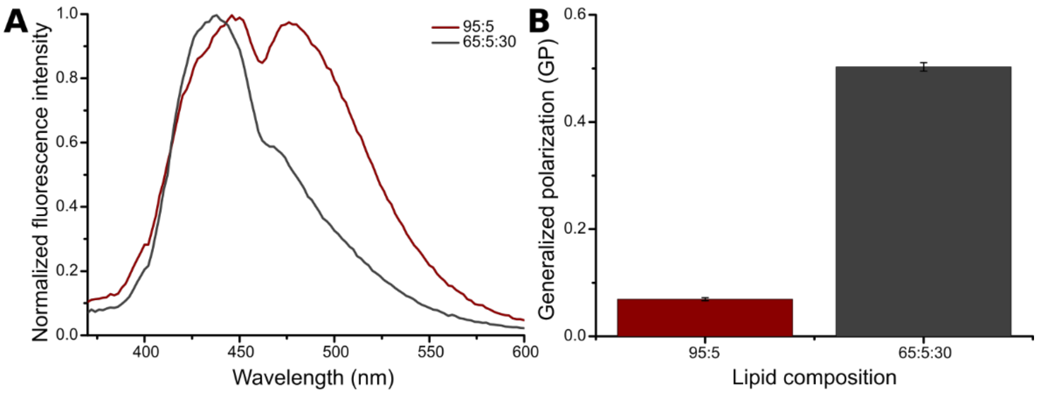

Figure S1. (A) Normalized mean fluorescence intensity of Laurdan emission spectra from vesicles with POPC/MPB-PE (95:5) (red) and POPC/MPB-PE/Ch (65:5:30) (black) containing 0.3 mol% Laurdan. Excitation wavelength 350 nm, n = 2. (B) Generalized polarization (GP) values calculated from the Laurdan fluorescence intensities in A.

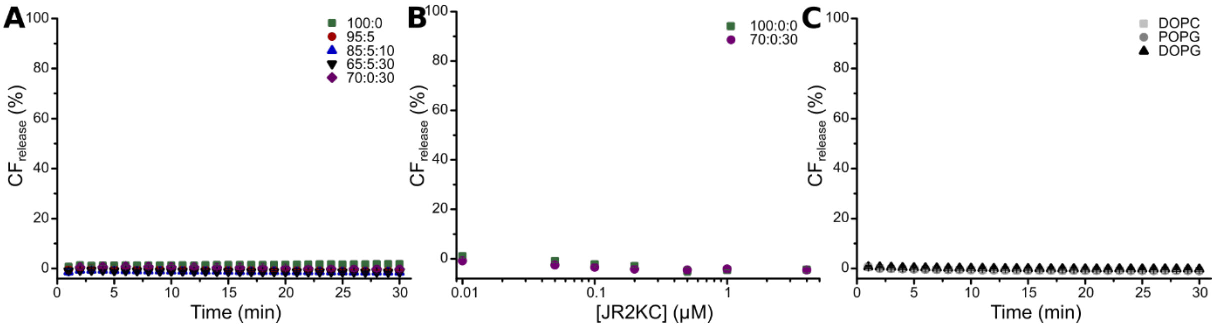

Figure S2. (A) CF release kinetics for LUVs with POPC/MPB-PE/Ch at the ratios (100:0:0), (95:5:0), (85:5:10), (65:5:30) and (70:0:30) in the absence of JR2KC. Lipid concentration: 40 μM, n ≥ 2. (B) Total CF release after 30 min from LUVs with 0 mol% MPB-PE, with or without cholesterol (30 mol%), after addition of JR2KC (0.01 – 4 μM). Total lipid concentration: 40 μM, n ≥ 2. (C) CF release kinetics of LUVs with POPC/MPB-PE/Ch/(DOPC/POPG/DOPG) (65:2.5:30:2.5) in the absence of JR2KC. Lipid concentration: 40 μM, n =3.

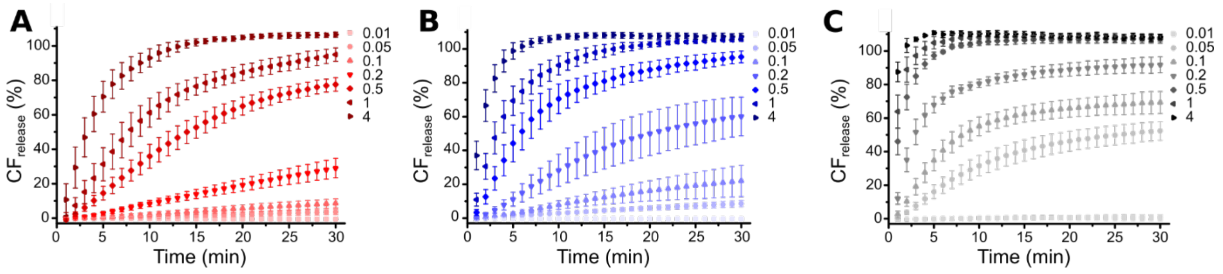

Figure S3. Kinetics for the CF release showed in Figure 1B, measured every minute for 30 min after addition of JR2KC (0.01 – 4 μM) to LUVs with A) POPC/MPB-PE (95:5), B) POPC/MPB-PE/Ch (85:5:10) and C) POPC/MPB-PE/Ch (65:5:30). Total lipid concentration: 40 μM. Each data point is the average of n = 4.

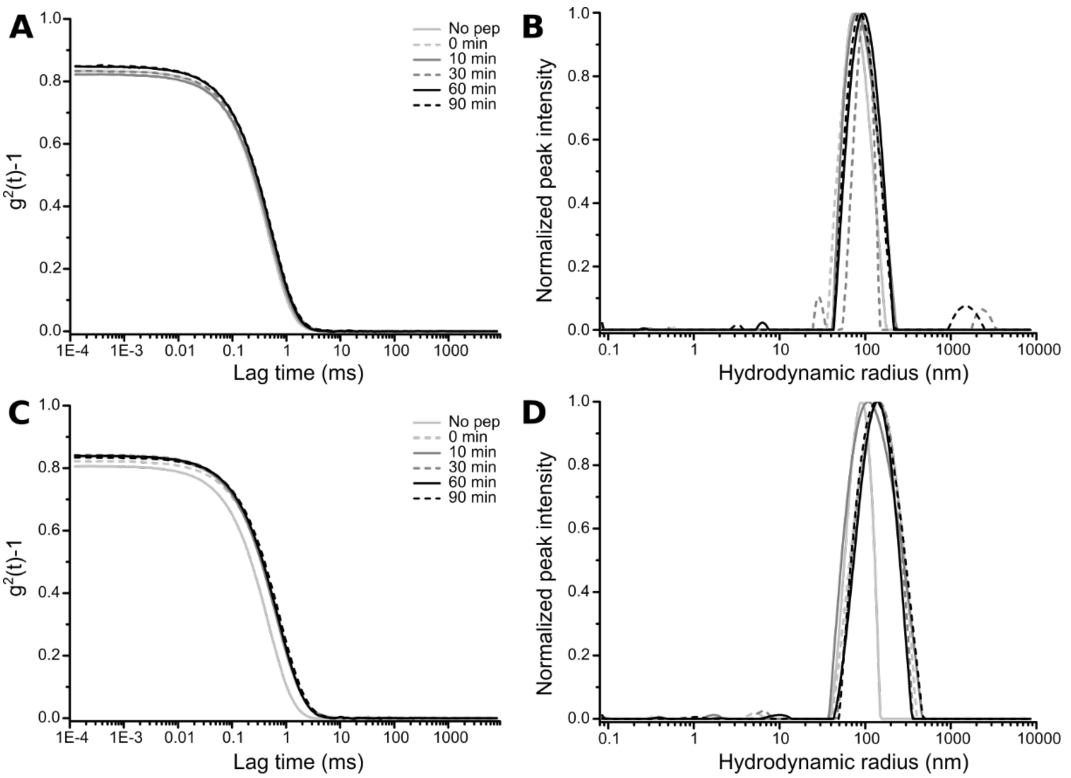

Figure S4. DLS correlation functions (A, C) and size distributions (B, D) of LUVs (40 μM) comprised of POPC/MPB-PE (95:5) (A, B) and POPC/MPB-PE/Ch (65:5:30) (C, D) recorded during 90 min after addition of 4 μM JR2KC.

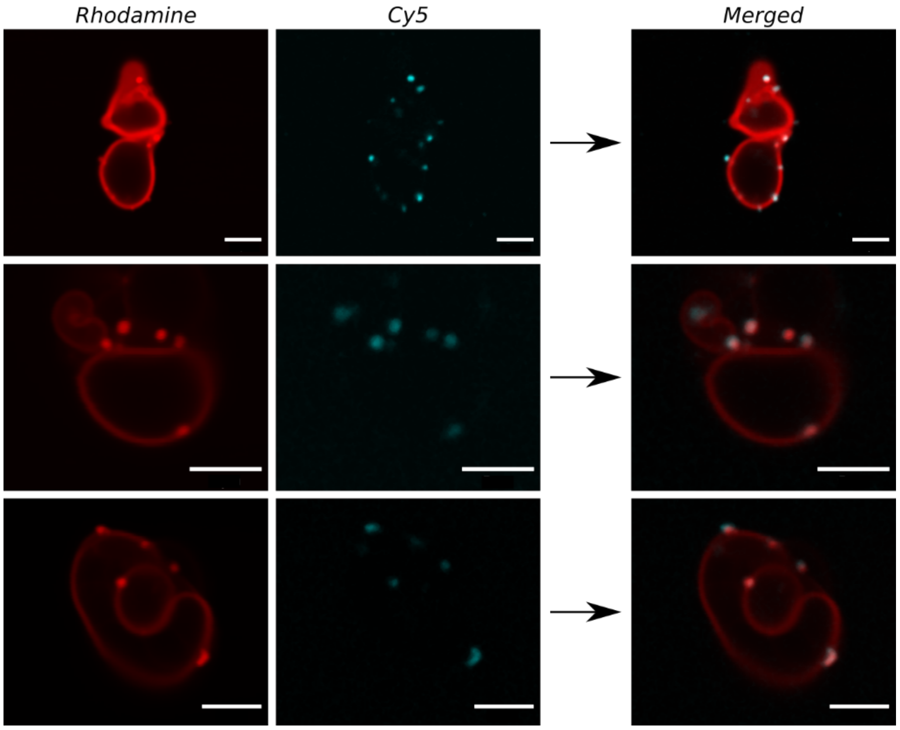

Figure S5. Representative confocal fluorescence microscopy images of GUVs composed of POPC/MPB-PE/Ch/Liss Rhod-PE (65:5:30:0.5) incubated with JR2KC-Cy5. Columns as indicated show rhodamine fluorescence (λ_{ex} = 561 nm and λ_{em} = 570-600 nm) and Cy5 fluorescence (λ_{ex} = 633 nm and λ_{em} = 650-690 nm). Scale bars: 5 µm.

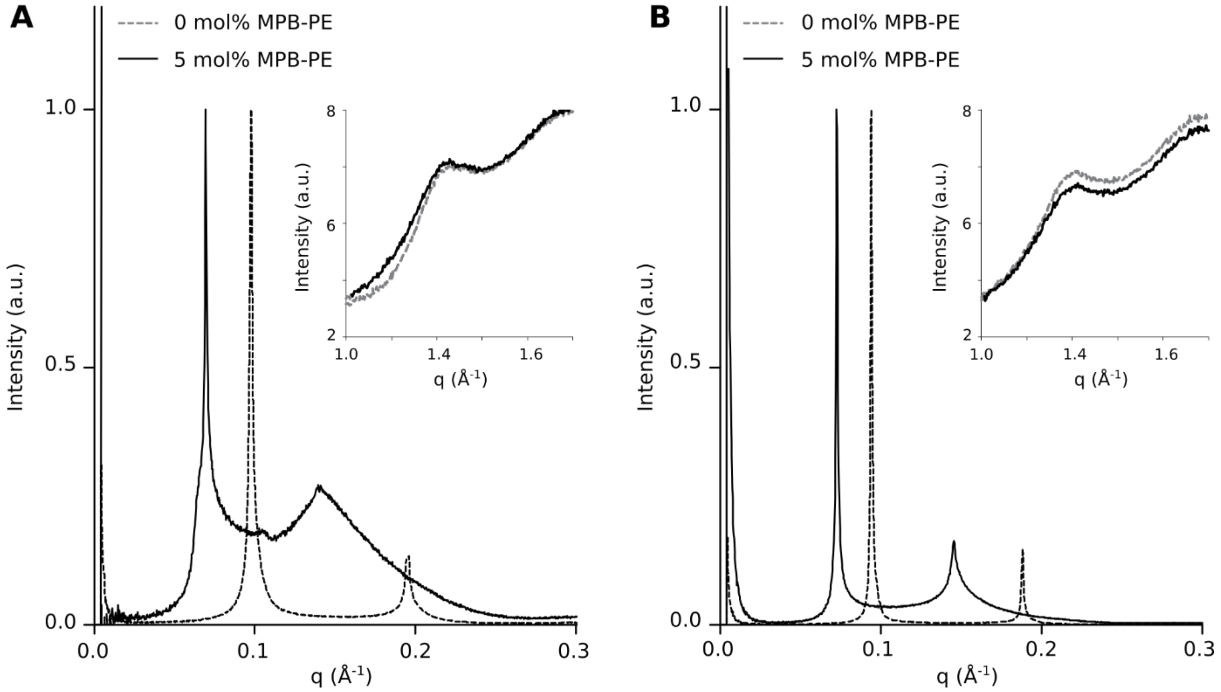

Figure S6. SAXS and WAXS (inset) scattering patterns showing the effect of adding 5 mol% MPB-PE to (A) POPC/MPB-PE (95:5) and (B) POPC/MPB-PE/Ch (65:5:30).

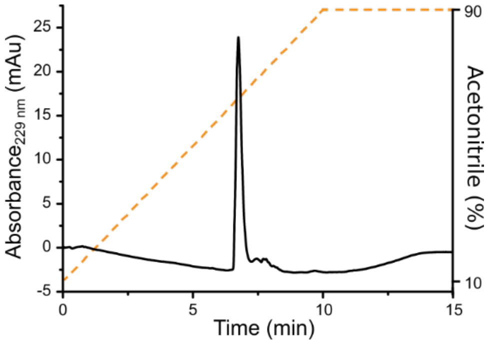

Figure S7. Analytical HPLC chromatogram of JR2KC$_{ref}$ showing peptide purity. Orange dashed line indicates the aqueous gradient of acetonitrile used.
